# Anti-tumour activity of tachykinin NK_1_receptor antagonists on human glioma U373 MG xenograft

**DOI:** 10.1054/bjoc.1999.0946

**Published:** 2000-01-18

**Authors:** C Palma, M Bigioni, C Irrissuto, F Nardelli, C A Maggi, S Manzini

**Affiliations:** Department of Pharmacology, Menarini Ricerche S.p.A., Via Tito Speri 10, Pomezia, RM 00040, Italy; Menarini Ricerche S.p.A., Via F. Rismondo 12 A, Firenze, 50131, Italy

**Keywords:** glioma, tumour xenograft, substance P, tachykinin NK_1_receptor antagonist, anti-tumour activity

## Abstract

Astrocytes harbour functional receptors to many neurotransmitters, including substance P (SP), an undecapeptide belonging to the tachykinin family of peptide transmitters. SP activates malignant glial cells to induce cytokine release and proliferation, both responses being relevant for tumour progression. In tumours developed in nude mice transplanted subcutaneously (s.c.) to U373 MG human glioma cells, the presence of SP was observed at immunohistochemistry. Although the administration of exogenous SP did not significantly affect the size or development of U373 MG xenograft, a role of SP in supporting glioma progression in vivo was highlighted by the tumour growth inhibition induced by highly specific and selective human tachykinin NK_1_receptor antagonists (MEN 11467 and MEN 11149). The anti-tumour activity of MEN 11467 was observed both with s.c. or intravenous treatments and was partially reverted by the concomitant administration of exogenous SP. Doxorubicin did not show any activity in controlling U373 MG growth in this in vivo model. A novel therapeutic approach to treat malignant gliomas with tachykinin NK_1_receptor antagonists is suggested by these findings. © 2000 Cancer Research Campaign


*British Journal of Cancer* (2000) **82**(2), 480–487
© 2000 Cancer Research Campaign
Article no. bjoc.1999.0946

### Anti-tumour activity of tachykinin $NK_1$ receptor antagonists on human glioma U373 MG xenograft

**C Palma[1], M Bigioni[1], C Irrissuto[1], F Nardelli[1], CA Maggi[2] and S Manzini[1]**

[1] Menarini Ricerche S.p.A., Department of Pharmacology, Via Tito Speri 10, 00040 Pomezia, RM, Italy; [2]Menarini Ricerche S.p.A., Via F. Rismondo 12 A, 50131 Firenze, Italy

**Summary** Astrocytes harbour functional receptors to many neurotransmitters, including substance P (SP), an undecapeptide belonging to the tachykinin family of peptide transmitters. SP activates malignant glial cells to induce cytokine release and proliferation, both responses being relevant for tumour progression. In tumours developed in nude mice transplanted subcutaneously (s.c.) to U373 MG human glioma cells, the presence of SP was observed at immunohistochemistry. Although the administration of exogenous SP did not significantly affect the size or development of U373 MG xenograft, a role of SP in supporting glioma progression in vivo was highlighted by the tumour growth inhibition induced by highly specific and selective human tachykinin $NK_1$ receptor antagonists (MEN 11467 and MEN 11149). The anti-tumour activity of MEN 11467 was observed both with s.c. or intravenous treatments and was partially reverted by the concomitant administration of exogenous SP. Doxorubicin did not show any activity in controlling U373 MG growth in this in vivo model. A novel therapeutic approach to treat malignant gliomas with tachykinin $NK_1$ receptor antagonists is suggested by these findings. © 2000 Cancer Research Campaign

**Keywords:** glioma; tumour xenograft; substance P; tachykinin $NK_1$ receptor antagonist; anti-tumour activity

Malignant astrocytomas or glioblastomas represent the most common type of primary brain tumour in adults (Salcman and Kaplan, 1986; Hochberg and Pruitt, 1987). High-grade malignant gliomas are inevitably lethal neoplasms and the median survival of patients treated with standard cytoreductive surgery and post-operative radiotherapy is in the range of 1 year. Likewise chemotherapy is of limited effectiveness in the treatment of these tumours and other therapeutic approaches must be explored. Glioblastomas are sensitive to several hormones and growth factors. The presence of receptors for epidermal growth factor, platelet-derived growth factor, insulin-like growth factor-I/II as well as for glucocorticoid, androstenedione and progesterone in malignant gliomas, has been described (Shapiro et al, 1995). Recently, neurotransmitters/neuropeptides have also been found to regulate astrocytoma growth (Sharif, 1998).

Substance P (SP), an undecapeptide of the tachykinin family of neuropeptides, is released both in the central and peripheral nervous systems, playing a well established role in neuronal transmission, vasodilatation and motor responses (Maggi et al, 1993). Tachykinins exert their actions through activation of G-protein coupled receptors, labelled $NK_1$, $NK_2$, $NK_3$; SP is the preferential endogenous ligand for the tachykinin $NK_1$ receptor (Maggi et al, 1993; Otsuka and Toshioka, 1993). In various astrocytic/glial brain tumour-derived cell lines, the presence of tachykinin $NK_1$ receptor strictly correlates with the effect of SP and/or NKA in increasing DNA synthesis and cellular proliferation (Luo et al, 1996; Sharif et al, 1996; Palma et al, 1999*a*). In addition, SP can control many other glial responses such as taurine release, secretion of various cytokines (e.g. interleukin (IL)-6, IL-8, transforming growth factor-β, leukaemia inhibitory factor, granulocyte–macrophage colony stimulating factor) which are thought to be relevant for glioma progression (Gitter et al, 1994; Palma et al, 1995; Palma and Manzini, 1998). In a large number of these studies, the human astrocytoma grade III U373 MG cell line, which expresses a high level of functional tachykinin $NK_1$ receptor (Lee et al, 1992; Palma et al, 1999*b*) but not tachykinin $NK_2$ or $NK_3$ receptors (Heuillet et al, 1993), were used.

The role of SP in glioma activities may be ascribed to a pathological use of normal signal transduction pathways occurring in reactive astrocytes. In fact, SP activates phospholipase C and stimulates the release of IL-6 and prostaglandin $E_2$ from human fetal astrocytes in culture (Palma et al, 1997), and there is evidence for an up-regulation of this receptor by reactive proliferating astrocytes after transection of the optic nerve (Mantyh et al, 1989). Moreover, SP-immunoreactive astrocytes have been observed in multiple sclerosis plaques (Kostyk et al, 1989) and in the forebrains of human infants (Michel et al, 1986). Interestingly, the involvement of tachykinin $NK_1$ receptor and SP in glioma progression was not only supported by the functional in vitro studies on human glioma cell lines but also by some clinical evidence. In fact, astrocytoma and glioblastoma contain significant levels of high affinity receptors for SP and their increase in expression correlate with the most malignant phenotype (Henning et al, 1995). Moreover, the presence of the SP itself has been documented in human malignant glioma tissues (Allen et al, 1985).

In the last decades a wide range of antagonists highly specific and selective for human tachykinin $NK_1$ receptors have been described with heterogeneous chemical structures and types of pharmacological antagonism (Quartara and Maggi, 1998). MEN 11467 (1R,2S)-2N-[1(H)indol-3-yl-carbonyl]-1-N-{$N^{\alpha}$(p-tolylacetyl)-$N^{\alpha}$(methyl)-D-3-(2-naphthyl)alanyl}diaminocyclohexane) and MEN 1149 (2-(2-naphthyl)-1-N-{(1R,2S)-2-N-[1(H)indol-3-yl-carbonyl]aminocyclohexanecarbonyl}-1-[N′-

*Received 6 May 1999*
*Received 7 July 1999*
*Accepted 9 July 1999*

*Correspondence to:* C Palma methyl-N′-(4-methylphenylacetyl)]diaminoethane are extremely potent human tachykinin $NK_1$ receptor antagonists. In radioligand binding studies, conducted with [$^3$H]SP on intact U373 MG cells, these antagonists interacted with the receptor in an irreversible manner and showed high affinity for the receptors with $K_i$ values of about 1 nM (Cirillo et al, 1998*a*; Palma et al, 1999*b*). On the contrary they had virtually no affinity ($K_i$ values ≥ 10 000 nM) for rat or murine tachykinin $NK_1$ receptors (Cirillo et al, 1998*a*, 1998*b*). Receptor blockade by both antagonists resulted in a powerful and complete inhibition of functional U373 MG responses induced by SP, such as accumulation of the second messenger inositol monophosphate or interleukin-6 release (Palma et al, 1999*a*, 1999*b*). Moreover, these antagonists were more potent in inhibiting late rather than early phases of SP-induced inositol monophosphate accumulation and their antagonisms were enhanced by drug preincubation and barely affected by removal of unbound drug from the external medium, suggesting that they bound in a tight manner to the receptor (Palma et al, 1999*b*). Interestingly, MEN 11467 was able to completely inhibit the SP-induced proliferation, both in terms of DNA synthesis and increase of cell number, in U373 MG cells without affecting both the basal or IL-1β-stimulated cell growth (Palma et al, 1999*a*).

These findings prompted us to investigate the effects of tachykinin $NK_1$ receptor antagonists on the growth of human brain tumours in in vivo mouse glioma xenograft model.

### MATERIALS AND METHODS

### Drugs

The tachykinin $NK_1$ receptor antagonists, MEN 11467 ((1R,2S)-2N[1(H)indol-3-yl-carbonyl]-1-N-{N$^\alpha$(p-tolylacetyl)-N$^\alpha$(methyl)-D-3-(2-naphthyl)alanyl}diaminocyclohexane; MW = 600.8) (Cirillo et al, 1998*a*; Palma et al, 1999*b*) and MEN 11149 (2-(2-naphthyl)-1-N-{(1R,2S)-2-N-[1(H)indol-3-yl-carbonyl]aminocyclohexane carbonyl}-1-[N′-methyl-N′-(4-methylphenylacetyl)]diaminoethane; MW = 600) (Cirillo et al, 1998*b*), were synthesized at the Chemistry Department of Menarini Ricerche, Pomezia, Italy (Figure 1). For the purposes of this study, these antagonists were solubilized in dimethyl sulphoxide (DMSO) and diluted in saline containing 6% Tween-80. SP was purchased from Calbiochem-Novabiochem AG (Laufelingen, Switzerland) solubilized in water and diluted in saline for administration to mice. Doxorubicin was purchased as a pharmaceutical formulation from Pharmacia Upjohn (Nerviano, Milan, Italy) and freshly prepared before use.

### Animals

Female athymic *nu/nu* nude mice, 5–7 weeks old were purchased from Harlan Nossan S.r.l. (Corezzana, MI, Italy), maintained in microisolator cages and supplied with sterile materials.

### Cell culture

The human glioma cell line, U373 MG (astrocytoma grade III) was obtained from the American Type Culture Collection (Rockville, MD, USA). The human ovarian carcinoma cell line A2780 was a kind gift of Dr Franco Zunino, Istituto Nazionale Tumori, Milan, Italy. Cells were cultured in RPMI-1640 medium containing 10% heat-inactivated (65°C, 30 min) fetal bovine serum with 5 mM HEPES, 2 mM L-glutamine, 100 U ml$^{-1}$ of penicillin and 100 μg ml$^{-1}$ of streptomycin at 37°C in an atmosphere of 5% carbon dioxide. All reagents were purchased from Gibco Laboratories (Grand Island, NY, USA).

### Xenograft model in athymic mice

Tumour xenografts were obtained by subcutaneous (s.c.) injection of $2 \times 10^7$ U373 MG cells or $10^7$ A2780 cells into the right flanks of female nude mice. Five days after tumour cell inoculation U373 MG or A2780 tumour-bearing mice were randomly divided into different groups (six animals per group) and treated intravenously (i.v.) or s.c. as reported in Results. In local treatments all the s.c. injections of MEN 11467 were sited at about 5 mm from the tumour. MEN 11467 or MEN 11149 were administered at doses that have been previously shown to produce a complete blockade of tachykinin $NK_1$-mediated response (bronchoconstriction) elicited by a systemic administration of Sar$^9$SP, a selective agonist of $NK_1$ tachykinin receptor in guinea pig (Cirillo et al, 1998*a*, 1998*b*).

The tumours were measured in two diameters and the tumour volume (TV) was calculated by the formula: TV = length (mm) × width$^2$ (mm)/2 = mm$^3$ (Geran et al, 1972). Optimal tumour volume inhibition per cent (TVI%) in treated over control mice was evaluated as: 100-(TV treated/TV control × 100) (Geran et al, 1972).

The doubling time in days was measured from a best-fit straight line of the control tumours in exponential growth (range, 100 ÷ 400 mm$^3$).

The non-parametric Mann–Whitney test was used for statistical comparison of tumour volume in mice treated with $NK_1$ antagonist

MEN 11149

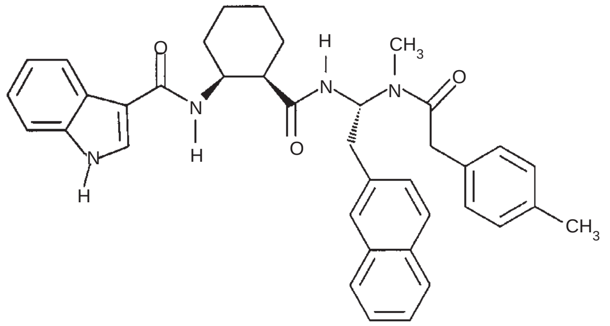

MEN 11467

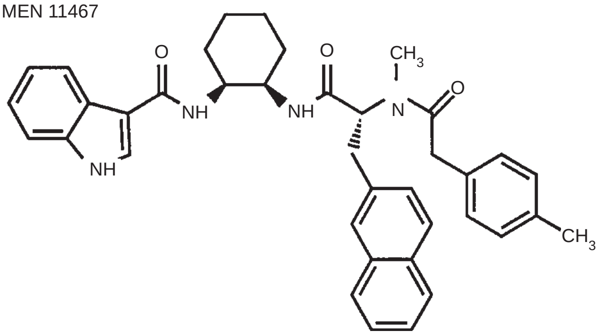

**Figure 1** Chemical structures of MEN 11149 and MEN 11467

versus vehicle treated mice. All animal work was carried out in accordance with UKCCCR guidelines for the welfare of animals in experimental neoplasia.

### Histological and immunohistochemical studies

At necropsy, tumour masses were preserved in 10% buffered formol saline. The samples were then dehydrated, embedded in paraffin wax and sections were cut at 5-μm thickness. Haematoxylin and eosin stain was performed in order to evaluate necrotic areas and inflammatory reactions.

Sections were also stained for SP and glial fibrillary acidic protein (GFAP) with the indirect immunoperoxidase technique using the kit specific for rabbit or mouse primary antibodies (LSAB/HRP, K681 DAKO, Glostrup, Denmark). Specific polyclonal rabbit antibodies for SP or GFAP (SIGMA, Chemical Co., St Louis, MO, USA) were applied at 1:400 dilution for 2 h at r.t. or at 1:50 dilution for 15 min at r.t. respectively. At the end of immunoperoxidase procedures, sections were lightly counterstained with haematoxylin for 1 min and automatically mounted with Tissue Tek Coverslipper, Bayer mod. 4765.

### RESULTS

### Presence of SP-like immunoreactivity in tumoural area of U373 MG xenograft in nude mice

Histologically, the U373 MG s.c. xenografted in nude mice retain some features of a human anaplastic astrocytoma grade III according to classification of this cell line. A consistent tumour cellularity, anaplasia, presence of mitotic events and necrosis were observed. Immunoreactivity of GFAP, a marker of astrocytic differentiation, was present in the cytoplasm of a proportion of tumour cells. A gain in tumoural volume was correlated with an increase in necrotic area. Infiltration of inflammatory cells, mainly lymphocytes, neutrophils and macrophages was present in the peripheral area of the tumour and in the perinecrotic zones.

Characteristic patterns of specific GFAP or SP-like immunoreactivity in a tumour explant obtained 40 days after U373 MG implant (vehicle group) are shown in Figure 2. On inspection at low magnification (25×), the presence of SP-like immunoreactivity was observed in the peritumoural skin as well as in various areas inside the tumour mass, especially in the periphery (Figure 2A). On the other hand, GFAP was localized only in specific areas

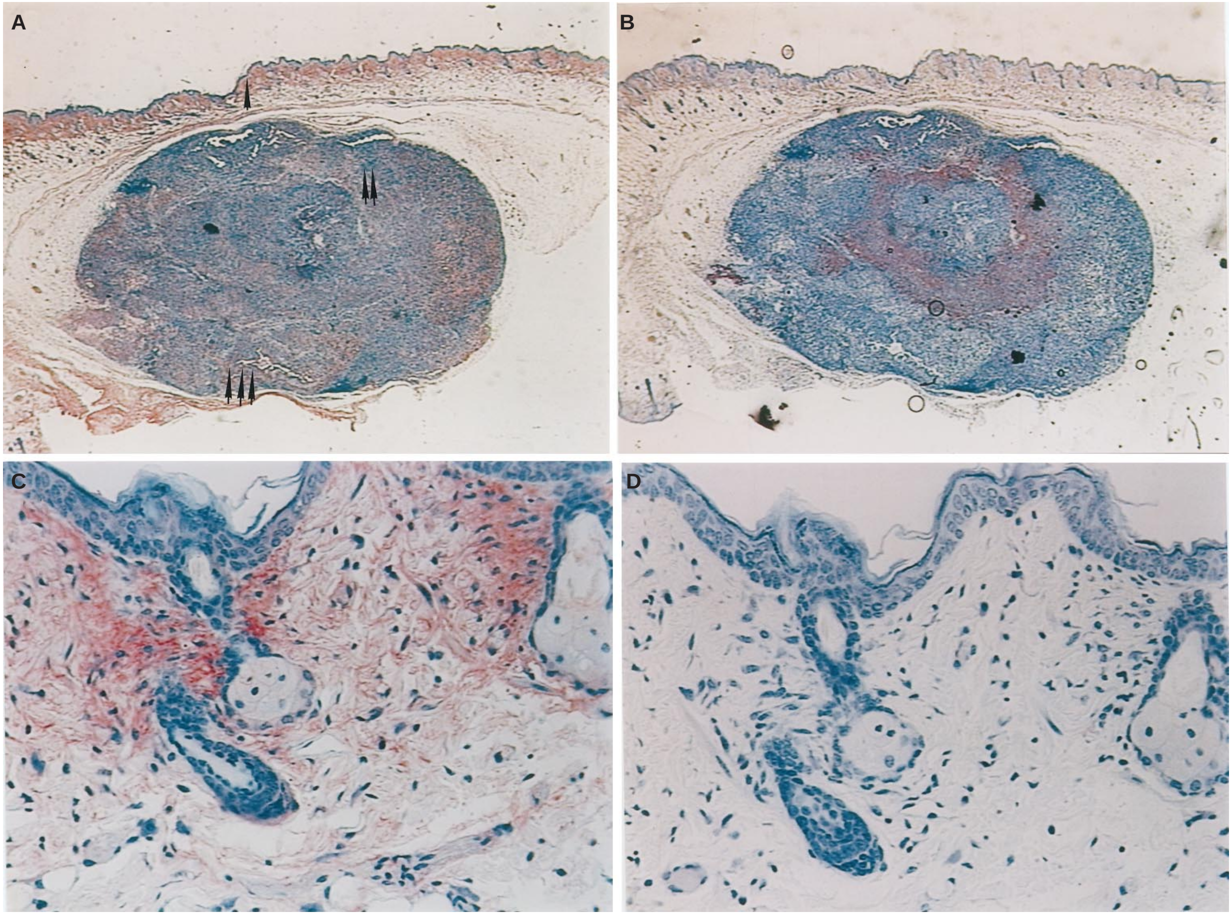

**Figure 2** Photomicrograph of cryostat U373 MG xenograft sections immunostained with antibodies to SP or GFAP by indirect immunoperoxidase and counterstained with haematoxylin. (**A, B**) SP and GFAP staining, respectively, at low magnification (25×); (**C, D**) a portion of murine skin (one arrow in panel A) immunostained with SP and GFAP antibodies respectively (200×); (**E, F**) a portion of internal tumour section (two arrows in panel **A**) immunostained with SP and GFAP antibodies, respectively (200×); (**G, H**) a portion of peripheral tumour section (three arrows in panel **A**) immunostained with SP and GFAP antibodies, respectively (200×)

 © 2000 Cancer Research Campaign

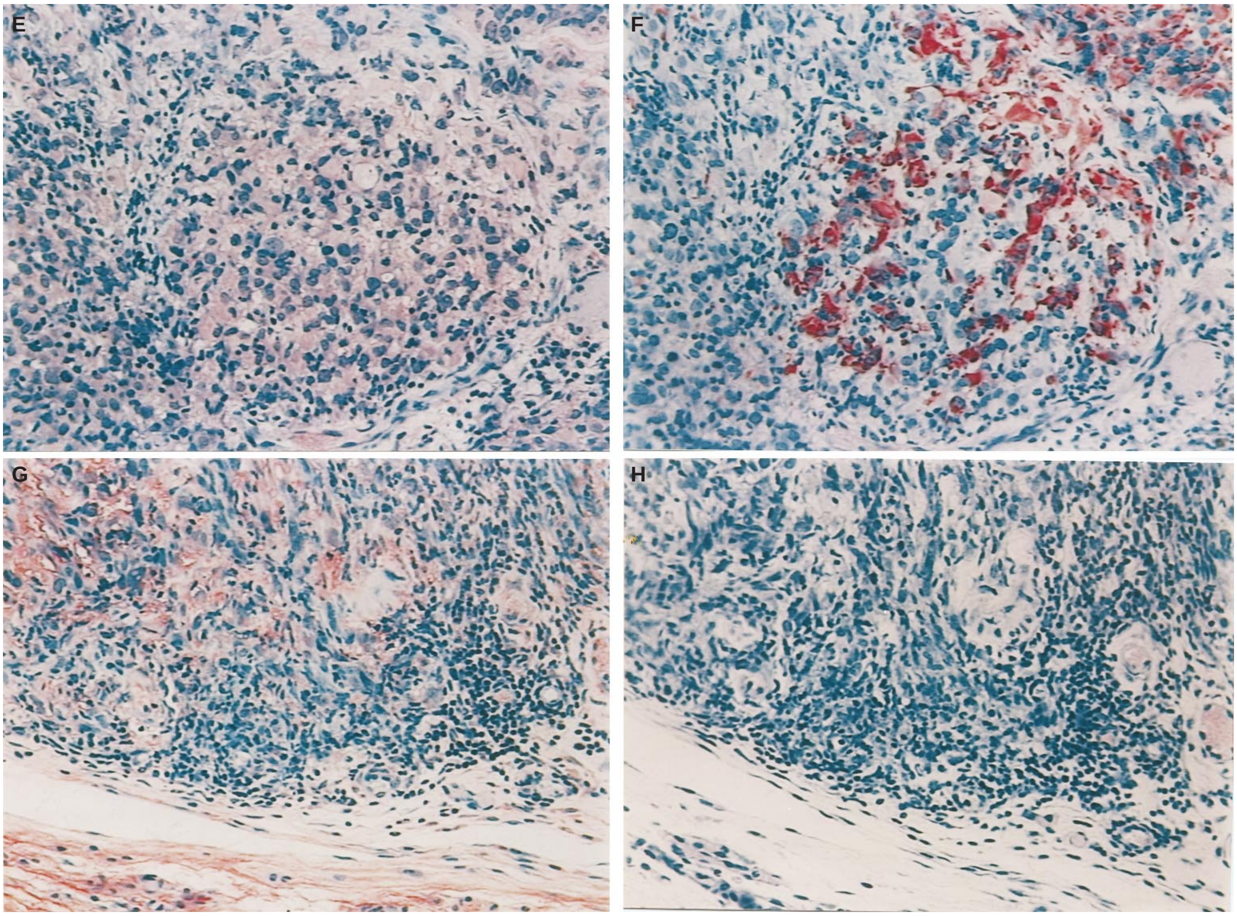

**Figure 2(cont.)**

inside the tumour (Figure 2B). At higher magnification (200×), there was evidence that GFAP staining was restricted only to a proportion of differentiated astrocytoma cells (Figure 2F). GFAP immunoreactivity was not observed in the anaplastic undifferentiated U373 MG cells or in the murine cells (Figures 2D, H). The nervous endings present in the dermal tissue (especially around the hair bulb, the sebaceous glands (Figure 2C) and in the muscle fibres (Figure 2G)) of the mouse peritumoural skin showed a remarkable positivity for SP-like immunoreactivity. In a peripheral tumoural area, a strong SP-like immunoreactivity was also observed, although not clearly expressed by tumour cells (Figure 2G). In contrast, specific SP staining was not detected in the more central tumour zone (Figure 2E). SP-like immunoreactivity was also found in the peritumoural skin and peripheral tumour areas of the human ovarian carcinoma A2780 xenograft (data not shown). Since SP staining is absent in U373 MG cells cultured in vitro (data not shown), the reported results suggest that the presence of SP in the xenografted U373 MG tumour mass originates from the host.

SP-immunoreactivity inside the U373 MG tumour xenograft, with the characteristics described above, was found in all the tumour explants ($n$ = 30) analysed independently of the tumoural volume, the animal treatments ($NK_1$ receptor antagonist and/or SP exogenous somministration, see below) and the phase of tumoural growth (data not shown).

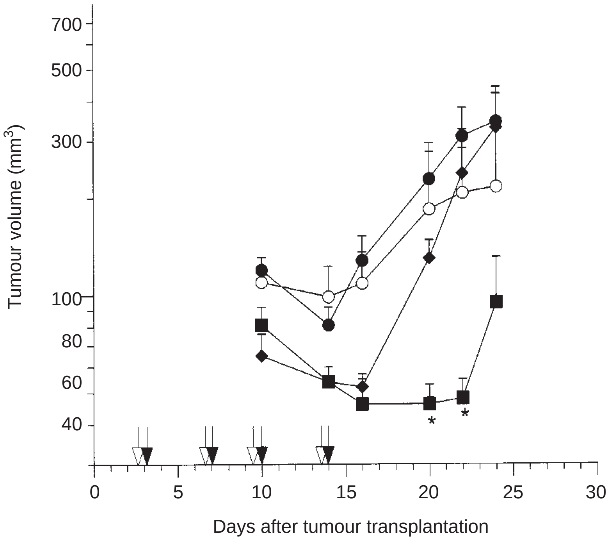

**Figure 3** Response of U373 MG human glioma during the following s.c. treatment: saline (○); SP (1 μmol $kg^{-1}$) (●); MEN 11467 (1 μmol $kg^{-1}$) (■); MEN 11467 (1 μmol $kg^{-1}$) + SP (1 μmole $kg^{-1}$) (administered simultaneously) (◆). Arrows indicate the days of treatment. Each point on the graph indicates the average volume of six tumours ± s.e.m. Differences from control values were determined by the non-parametric Mann–Whitney test and are indicated as follows: *$P \leq 0.05$

*© 2000 Cancer Research Campaign*

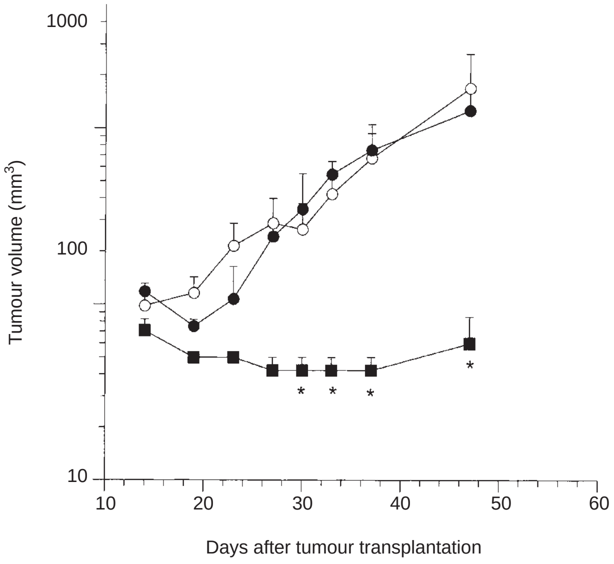

**Figure 4** Response of U373 MG human glioma during the following s.c. treatment: saline (○); SP (0.4 μmol kg$^{-1}$) three times a week until the end of experiment (●); MEN 11467 (1.7 μmol kg$^{-1}$) five times a week and SP (0.4 μmol kg$^{-1}$) three times a week until the end of experiment (■). Each point on the graph indicates the average volume of six tumours ± s.e.m. Differences from control values were determined by the non-parametric Mann–Whitney test and are indicated as follows: $^*P \leq 0.05$

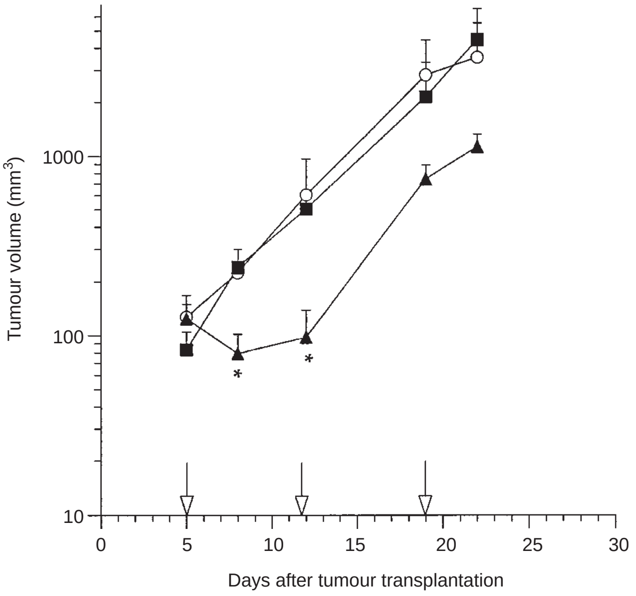

**Figure 5** Response of A2780 human ovarian carcinoma during the following treatment: saline (○); MEN 11467 (1.7 μmol kg$^{-1}$ s.c.) five times a week until the end of the experiment (■), doxorubicin (7 mg kg$^{-1}$ i.v.) (▲). Arrows indicate the days of doxorubicin administration. Each point on the graph indicates the average volume of six tumours ± s.e.m. Differences from control values were determined by the non-parametric Mann–Whitney test and are indicated as follows: $^*P \leq 0.05$

### Effect of tachykinin $NK_1$ receptor antagonists on U373 MG xenograft growth in nude mice

To better define the effects of the neuropeptide SP as a tumour glioma growth factor in vivo, two different schedules of s.c. SP administration were assayed in nude mice transplanted s.c. to U373 MG cells. As shown in Figures 3 and 4, both treatments (1 μmole kg$^{-1}$ twice a week for 2 weeks or 0.4 μmol kg$^{-1}$ three times a week for 6 weeks) produced negligible or only slight stimulant effects on tumour growth, suggesting that the addition of exogenous SP was not necessary for supporting U373 MG in vivo growth. A role of SP in U373 MG in vivo growth was highlighted by the anti-tumour activity obtained with SP receptor antagonists. The pseudopeptide, MEN 11467, which possesses a high affinity for the human tachykinin $NK_1$ receptor but does not bind the murine $NK_1$ receptor (Cirillo et al, 1998*a*), was used to block $NK_1$ receptors expressed in U373 MG cells. As shown in Figure 3, s.c. treatment with MEN 11467 (1 μmol kg$^{-1}$ twice weekly for 2 weeks) resulted in a temporary growth arrest of the U373 MG xenograft that lasted for about 10 days until the last MEN 11467 administration (TVI% = 56). Thereafter, the tumour started to regrow. MEN 11467 anti-tumour activity was partially reverted by the simultaneous administration of an equimolar dose of exogenous SP, suggesting the specificity of tachykinin $NK_1$ receptor activation in glioma growth (Figure 3).

Prolonged s.c. treatment with a higher MEN 11467 dose (1.7 μmol kg$^{-1}$ at five times a week for 6 weeks) completely inhibited the growth of U373 MG tumour for the entire length of the experiment, even following administration of a low exogenous SP dose (Figure 4). After 6 weeks, the tumour mass was not increased compared to the untreated control with TVI% = 96%. At the end of experiments there was no indication of toxicity following MEN 11467 treatment, either in terms of body weight loss or general toxicity (data not shown). Under the same experimental conditions, treatment with MEN 11467 (1.7 μmol kg$^{-1}$ s.c. daily for 3 weeks) did not inhibit the growth of A2780 (Figure 5).

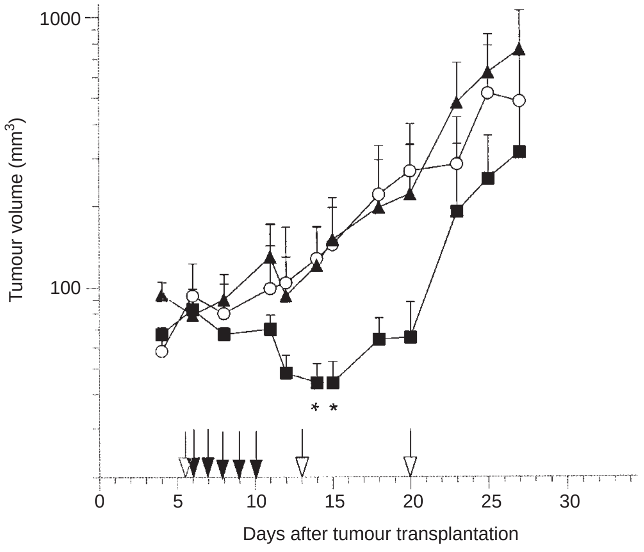

**Figure 6** Response of U373 MG human glioma during the following i.v. treatment: saline (○); doxorubicin (7 mg kg$^{-1}$) (▲); MEN 11467 (1 μmol kg$^{-1}$) (■). Empty arrows indicate the days of doxorubicin administration while solid arrows indicate the days of MEN 11467 administration. Each point on the graph indicates the average volume of six tumours ± s.e.m. Differences from control values were determined by the non-parametric Mann–Whitney test and are indicated as follows: $^*P \leq 0.05$

The anti-tumour activity of MEN 11467 was also observed in a systemic treatment. As shown in Figure 6, i.v. administration with

© 2000 Cancer Research Campaign

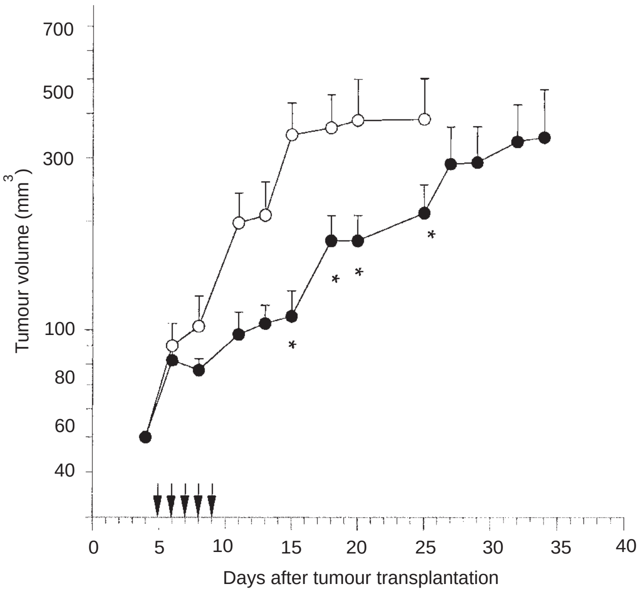

**Figure 7** Response of U373 MG human glioma during the following i.v. treatment: saline (○); MEN 11149 (1 μmol kg$^{-1}$) (●). Solid arrows indicate the days of MEN 11149 administration. Each point on the graph indicates the average volume of six tumours ± s.e.m. Differences from control values were determined by the non-parametric Mann–Whitney test and are indicated as follows: *$P \leq 0.05$

MEN 11467 (1 μmol kg$^{-1}$ once daily for 5 consecutive days) caused a 10-day latency in U373 MG growth in vivo (TVI% = 76). Similar results in controlling glioma proliferation in vivo were achieved using the same treatment schedule (1 μmol kg$^{-1}$ i.v. once daily for 5 consecutive days), with another specific and selective human $NK_1$ receptor antagonist, MEN 11149 (Cirillo et al, 1998b) (TVI% = 54) (Figure 7).

Interestingly, doxorubicin administered at its optimal i.v. weekly schedule (7 mg kg$^{-1}$ for 3 consecutive weeks) (Giuliani et al, 1981), did not show any anti-tumour activity on U373 MG xenografts (Figure 6).

### DISCUSSION

Astrocytes are responsible for controlling brain homeostasis through the activation of a variety of responses. Some neurotransmitters, including SP and NKA, are important astrocytic stimuli representing the link of bidirectional interactions between glial cells and neurons. The SP-induced release of soluble mediators and proliferation observed in normal astrocytes (Mantyh et al, 1989; Palma et al, 1997), may be one of the pathways used by malignant glial cells. We reported a direct role of SP in supporting the glioma growth and its control by tachykinin $NK_1$ receptor blockade in an in vivo model. Nude mice transplanted s.c. with U373 MG cells developed a tumour retaining several features of a human anaplastic astrocytoma grade III. The murine skin, rich in nervous fibres containing SP, supplied an endogenous source of this neuropeptide. Thus the U373 MG xenograft in nude mice was a suitable in vivo model to verify the role of tachykinin $NK_1$ receptor antagonists in controlling glioma growth.

Blockade of tachykinin $NK_1$ receptors expressed in U373 MG cells was performed using the tachykinin $NK_1$ receptor antagonist MEN 11467 which endows high affinity for human $NK_1$ receptor subtypes but does not bind murine $NK_1$ receptors (Cirillo et al, 1998*a*). In in vitro assays, MEN 11467 completely inhibited SP-triggered U373 MG proliferation and cytokine release leaving unaltered the basal or IL-1β-induced responses as a proof of its specificity (Palma et al, 1999*a*). Moreover, MEN 11467 showed a long-lasting inhibitory activity, probably due to its very slowly reversible binding to the human tachykinin $NK_1$ receptor (Palma et al, 1999*b*). In fact, a potent inhibition of MEN 11467 in SP-induced IP1 accumulation, even following its removal from the external medium, was observed in U373 MG cells (Palma et al, 1999*b*). Treatment with MEN 11467 was able to control U373 MG growth in vivo. Prolonged s.c. treatment (1.7 μmol kg$^{-1}$ five times a week for 6 weeks) with MEN 11467 inhibited the growth of U373 MG xenograft for at least 6 weeks. Both s.c. and i.v. administration of MEN 11467 (1 μmol kg$^{-1}$) for a short period (1 or 2 weeks) prevented the tumour growth for about 10 days after the last MEN 11467 administration. The inhibition of tumour proliferation is not irreversible and a continuous blockade of tachykinin $NK_1$ receptors is required.

The observed arrest of glioma xenograft growth seems to be exclusively dependent on the inhibition of SP-stimulated signal transduction in U373 MG cells. Being MEN 11467 or MEN 11149 highly selective for the human tachykinin $NK_1$ receptor, we suppose that these drugs did not antagonize the possible SP-induced responses on murine cells (e.g. endothelial cells) that may have facilitated U373 MG xenograft growth. In addition, general toxicity was not observed in mice following s.c. or i.v. treatment with MEN 11467 or MEN 11149, excluding that the glioma growth inhibition observed was due to toxic aspecific effects of these compounds. Moreover, treatment with MEN 11467 did not alter the growth of A2780 xenograft. This human ovarian carcinoma cell line, lacking in tachykinin $NK_1$ receptors and unresponsive to SP in terms of cell proliferation and DNA synthesis (our unpublished observation) was used as a proof to assess the specificity of tachykinin $NK_1$ receptor activation in glioma growth. In addition, the role of SP in U373 MG xenograft development was also supported by the partial reversion of MEN 11467 inhibitory effects obtained with the simultaneous administration of high doses of exogenous SP (1 μmol kg$^{-1}$).

These data suggest an involvement of tachykinins in supporting glioma progression, although it has to be taken into account the limitations of the in vivo model used. The microenvironment of a flank xenograft is very different to that of the central nervous system and in this model the tachykinin-triggered tumour proliferation may be relevant compared to other glioma growth factors. In addition the results were obtained with a single human glioma cell line, the U373 MG which is highly responsive to SP stimulation (Luo et al, 1996; Palma et al, 1999*a*). Although we cannot extrapolate the data obtained with U373 MG cells to other cell lines, the role of tachykinin $NK_1$ receptor activation in human gliomas is supported by further evidence. In addition to U373 MG cells, several human glioma cell lines express functional tachykinin $NK_1$ receptors (Ogo et al, 1996; Sharif, 1998; Palma et al, 1999*a*). Moreover, the presence of SP receptors in glioma cells is correlated with a responsiveness to SP in terms of increase in mitogenesis and cytokine release (Palma et al, 1999*a*). The soluble factors (cytokines, prostaglandins, taurine) induced by SP activation in glioma cells are themselves tumour growth factors and can influence the tumour cell–host interactions including depression of the immune response, angiogenesis and microenvironment modifications (Jennings et al, 1991; Munoz-Fernandes and Fresno, 1993; Gillespie, 1996). Heterogeneity of human gliomas regarding

© 2000 Cancer Research Campaign their SP responsiveness and consequently their different susceptibility to tachykinin $NK_1$ receptor antagonist treatment, may be dependent on the origin of the transformed astrocytes. Differences in expression of tachykinin $NK_1$ receptors and in SP responsiveness were observed in human embryonic spinal cord or myelecephalon/mesencephalon astrocytes (Palma et al, 1997). In addition, it would be interesting to discover if differentiated and more anaplastic glioma cells, present also in U373 MG xenograft ($GFAP^+$ and $GFAP^-$ cells, respectively) express the same amount of tachykinin $NK_1$ receptors and if their activation induces comparable responses.

The possible involvement of the neurotransmitter SP in gliomas is also supported by some clinical findings. In human brain biopsies of glioma patients, the presence of the neuropeptide SP as well as the presence of tachykinin $NK_1$ receptors have been observed (Allen et al, 1985; Henning et al, 1995). Nine out of 12 astrocytomas and 10 out of 10 glioblastomas were positive for SP receptors, with an increasing percentage of tachykinin $NK_1$ receptor expression in tumours possessing the most malignant phenotype (Henning et al, 1995). In addition, the expression of tachykinin $NK_1$ receptors in peritumoural and tumoural blood vessels (Henning et al, 1995) suggests a role of SP in facilitating tumour blood supply by virtue of its tachykinin $NK_1$ receptor-mediated angiogenic and vasodilator properties (Ziche et al, 1990).

Although we were not able to detect a direct SP production by U373 MG in vivo, astrocytes themselves may be a source of this neuropeptide. Cultured astrocytes express mRNA for preprotachykinin, the precursor of SP (Too et al, 1994), and SP immunoreactivity or mRNA have been localized in reactive astrocytes in white matter lesions of multiple sclerosis brains (Kostyk et al, 1989) and in a mouse model of axonal degeneration (Yamazaki et al, 1993). In addition, a glial-cell-line-derived neurotrophic factor enhanced biosynthesis of SP in striatal neurons (Humpel et al, 1996), suggesting a modulation of SP release by astrocytes.

The tachykinin $NK_1$ receptor antagonist efficacy in a glioma in vivo model is an important finding as, at present, no curative therapy is available for malignant gliomas. The median survival of glioma patients treated with standard cytoreductive surgery and post-operative radiotherapy or chemotherapy is in the range of 1 year. In nude mice transplanted s.c. to U373 MG, no significant reduction in final tumour volume was obtained with doxorubicin, one of the most clinically useful cytotoxic drugs. The lack of cytotoxic effects by doxorubicin in our glioma in vivo model may be due to the low growth kinetics of U373 MG tumour in nude mice (doubling time = 7 ÷ 8 days) and, especially, to the intrinsic resistance of U373 MG cells. These astrocytoma cells overexpress the multidrug resistance (*mdr1*) gene and its product, P-glycoprotein, as well as the multidrug resistance-associated protein (Walther et al, 1995; Masanao, 1998).

In conclusion, we have gathered evidence that SP, through tachykinin $NK_1$ receptor activation, participates in U373 MG development and growth in vivo. Besides the limitations of the model proposed regarding the glioma growth in a micro-environment very different from the central nervous system and the use of just one human cell line, this study introduces the novel idea of a therapeutic approach with tachykinin $NK_1$ receptor antagonists to treat malignant gliomas. The incomplete knowledge of the role of tachykinins in the human central nervous system does not allow, at present, to foresee the side effects of a prolonged treatment with tachykinin $NK_1$ receptor antagonists.

### ACKNOWLEDGEMENTS

We wish to thank Alberta Argentino-Storino for the histological and immunohistochemical studies performed on U373 MG and A2780 xenografts. We are grateful to Maria Grandi for her critical review of this manuscript and her helpful suggestions.

### REFERENCES

Allen JM, Hoyle NR, Yeats JC, Ghatei MA, Thomas DG and Bloom SR (1985) Neuropeptides in neurological tumours. *J Neuro-Oncol* **3**: 197–202

Cirillo R, Astolfi M, Ciucci A, Palma C, Parlani M, Lopez G, Conte B, Terracciano R, Fincham CI, Sisto A, Maggi CA and Manzini S (1998*a*) Pharmacology of MEN 11467, a potent, selective and orally effective pseudopeptide tachykinin NK-1 antagonist. *Naun Schmied Arch Pharmacol* **358**: R324

Cirillo R, Astolfi M, Conte B, Lopez G, Parlani M, Terracciano R, Fincham CI and Manzini S (1998*b*) Pharmacology of the peptidomimetic, MEN 11149, a new potent, selective and orally effective tachykinin NK1 receptor antagonist. *Eur J Pharmacol* **341**: 201–209

Geran RI, Greenberg NH, MacDonald MM, Schumacher AM and Abbott BJ (1972) Protocols for screening chemical agents and natural products against animal tumors and other biological systems. *Cancer Chemother Rep* **3**: 1–88

Gillespie GY (1996) Cytokines as modulators of malignant glioma progression. In: *Cytokines and the CNS*, Ransohoff RM and Beneviste EM (eds), pp. 269–286. CRC Press: Boca Raton

Gitter BD, Regoli D, Howbert JJ, Glasebrook AL and Waters DC (1994) Interleukin-6 secretion from human astrocytoma cells induced by substance P. *J Neuroimmunol* **51**: 101–108

Giuliani FC, Karimullah AZ and Kaplan NO (1981) Therapeutic response of human tumor xenografts in athymic nude mice to doxorubicin. *Cancer Res* **41**: 325–335

Henning IM, Laissue JA, Horisberger U and Reubi JC (1995) Substance-P receptors in human primary neoplasms: tumoral and vascular localization. *Int J Cancer* **61**: 786–792

Heuillet E, Menager J, Faradin V, Flamand O, Bock M, Garret C, Crespo A, Fallourd AM and Doble A (1993) Characterization of a human NK1 tachykinin receptor in the astrocytoma cell line U373 MG. *J Neurochem* **60**: 868–876

Hochberg F and Pruitt A (1987) Neoplastic diseases of the central nervous system. In: *Harrison's Principles of Internal Medicine*, 11th edn. Braunwald E, Isselbacher KJ, Petersdorf RG, Wilson JD, Martin JB and Fauci AS (eds), pp. 1968–1980. McGraw-Hill: New York

Humpel C, Marksteiner J and Saria A (1996) Glial cell-derived neurotrophic factor enhances biosynthesis of substance P in striatal neurons in vitro. *Cell Tissue Res* **286**: 249–255

Jennings MT, Maciunas RJ, Carver R, Bascom CC, Juneau P, Misulis K and Moses HL (1991) TGFβ1 and TGFβ2 are potential growth regulators for low-grade and malignant glioma in vitro: Evidence in support of an autocrine hypothesis. *Int J Cancer* **49**: 129–139

Kostyk SK, Kowall NW and Hauser SL (1989) Substance P immunoreactive astrocytes are present in multiple sclerosis plaques. *Brain Res* **504**: 284–288

Lee CM, Tung WL and Young JD (1992) Tachykinin-stimulated inositol phospholipid hydrolysis and taurine release from human astrocytoma cells. *J Neurochem* **59**: 406–414

Luo W, Sharif TR and Sharif M (1996) Substance P-induced mitogenesis in human astrocytoma cells correlates with activation of the mitogen-activated protein kinase signaling pathway. *Cancer Res* **56**: 4983–4991

Maggi CA, Patacchini R, Rovero P and Giachetti A (1993) Tachykinin receptors and tachykinin receptor antagonists. *J Autonom Pharmacol* **13**: 23–93

Mantyh PW, Johnson DJ, Boehmer CG, Catton MD, Vinters HV, Maggio JE, Too HP and Vigna SR (1989) Substance P receptor binding sites are expressed by glia in vivo after neuronal injury. *Proc Natl Acad Sci USA* **86**: 5193–5197

Masanao M (1998) Expression of multidrug resistance-associated protein (MRP) in human gliomas. *Kanazawa Daigaku Juzen Igakkai Zasshi* **107**: 33–43

Michel JP, Sakamoto N, Bouvier R, Tommasi M and Pearson J (1986) Substance P-immunoreactive astrocytes related to deep white matter and striatal blood vessels in human brain. *Brain Res* **377**: 383–387

Munoz-Fernandes MA and Fresno P (1993) Involvement of nitric oxide on the cytokine induced growth of glial cells. *Biochem Biophys Res Commun* **194**: 319–325

© 2000 Cancer Research Campaign

Ogo H, Kuroyanagi N, Inoue A, Nishio H, Hirai Y, Akiyama M, DiMaggio DA, Krause JE and Nakata Y (1996) Human astrocytoma cells (U87 MG) exhibit a specific substance P binding site with the characteristics of an NK1 receptor. *J Neurochem* **67**: 1813–1820

Otsuka M and Toshioka K (1993) Neurotransmitter functions of mammalian tachykinins. *Physiol Rev* **73**: 229–308

Palma C and Manzini S (1998) Substance P induces secretion of immunomodulatory cytokines by human astrocytoma cells. *J Neuroimmunol* **81**: 127–137

Palma C, Urbani F and Manzini S (1995) Interleukin-6 production by U373 MG, a human astrocytoma cell line: different pathways involved in substance P and lipopolysaccharide activation. *J Neuroimmunol* **59**: 155–163

Palma C, Minghetti L, Astolfi M, Ambrosini E, Ceccherini Silberstein F, Manzini S, Levi G and Aloisi F (1997) Functional characterization of substance P receptors on cultured human spinal cord astrocytes: synergism of substance P with cytokines in inducing interleukin-6 and prostaglandin $E_2$ production. *Glia* **21**: 183–193

Palma C, Nardelli F, Manzini S and Maggi CA (1999*a*) Substance P activates responses correlated with tumor growth in human glioma cell lines bearing tachykinin $NK_1$ receptors. *Br J Cancer* **79**: 236–243

Palma C, Nardelli F and Manzini S (1999*b*) Correlation between binding characteristics and functional antagonism in human glioma cells by tachykinin $NK_1$ receptor antagonists. *Eur J Pharmacol* **374**: 435–443

Quartara L and Maggi CA (1998) The tachykinin $NK_1$ receptor: Part II. Distribution and pathophysiological role. *Neuropeptides* **32**: 1–49

Salcman M and Kaplan RS (1986) Intracranial tumors in adults. In: *Comprehensive Textbook of Oncology*, Moosa AR, Robson MC and Schimpff SC (eds), pp. 617–629. Williams and Wilkins: Baltimore

Shapiro WR, Shapiro JR and Walker RW (1995) Central nervous system. In: *Clinical Oncology*, Abeloff MD, Armitage JO, Licther AS and Niederhuber JE (eds), pp. 851–912. Churchill Livingstone: Edinburgh

Sharif M (1998) Mitogenic signaling by substance P and bombesine-like neuropeptide receptors in astrocytic/glial brain tumor-derived cell lines. *Int J Oncol* **12**: 273–286

Sharif TR, Luo W, Houghton PJ and Sharif M (1996) Substance K peptide induces mitogenesis by activating the mitogen-activated protein kinase signaling pathway through the substance P receptor (NK-1 subtype) in human astrocytoma. *Cell Pharmacol* **3**: 441–449

Too HP, Marriott DR and Wilkin GP (1994) Preprotachykinin-A and substance P receptor (NK1) gene expression in rat astrocytes in vitro. *Neurosci Lett* **195**: 57–60

Walther W, Stein U and Pfeil D (1995) Gene transfer of human TNF-alpha into glioblastoma cells permits modulation of mdr1 expression and potentiation of chemosensitivity. *Int J Cancer* **61**: 832–839

Yamazaki K, Moriya H, Ichihara N, Mitsushio H, Inagaki S and Kikuchi T (1993) Substance P-immunoreactive astrocytes in gracile sensory nervous tract of spinal cord in gracile axonal dystrophy mutant mouse. *Mol Chem Neuropathol* **20**: 1–20

Ziche M, Morbidelli L, Pacini M, Geppetti P, Alessandri G and Maggi CA (1990) Substance P stimulates neovascularisation in vivo and proliferation of cultured endothelial cells. *Microvasc Res* **40**: 264–278

*© 2000 Cancer Research Campaign*
